# pyhgf: A neural network library for predictive coding

**DOI:** 10.1371/journal.pcbi.1014340

**Published:** 2026-06-22

**Authors:** Nicolas Legrand, Lilian Weber, Peter Thestrup Waade, Anna Hedvig Møller Daugaard, Mojtaba Khodadadi, Nace Mikuš, Christoph Mathys

**Affiliations:** 1 Interacting Minds Centre, Aarhus University, Aarhus, Denmark; 2 Department of Psychiatry, University of Oxford, Oxford, United Kingdom; 3 Scuola Internazionale Superiore di Studi Avanzati (SISSA), Trieste, Italy; University of Cambridge, UNITED KINGDOM OF GREAT BRITAIN AND NORTHERN IRELAND

## Abstract

Bayesian models of cognition have gained considerable traction in computational neuroscience and psychiatry. Their scope is now expected to expand rapidly to artificial intelligence, providing general inference frameworks to support embodied, adaptable, and energy-efficient autonomous agents. A central theory in this domain is predictive coding, which posits that learning and behaviour are driven by hierarchical probabilistic inferences about the causes of sensory inputs. Biological realism constrains these networks to rely on simple local computations in the form of precision-weighted predictions and prediction errors. This can make this framework highly efficient, but its implementation comes with unique challenges on the software development side. Embedding such models in standard neural network libraries often becomes limiting, as these libraries’ compilation and differentiation backends can force a conceptual separation between optimisation algorithms and the systems being optimised. This critically departs from other biological principles such as self-monitoring, self-organisation, cellular growth, and functional plasticity. In this paper, we introduce pyhgf: a Python package backed by JAX and Rust for creating, manipulating, and sampling dynamic networks for predictive coding. We improve over other frameworks by enclosing the network components as transparent, modular, and malleable variables in the message-passing steps. The resulting graphs can implement arbitrary algorithms as belief propagation. Moreover, the transparency of core variables can also translate into inference processes that leverage self-organisation principles and express structure learning, meta-learning, or causal discovery as the consequence of network structural adaptation to surprising inputs. The main functions of the library are differentiable and seamlessly integrate into sampling or optimisation workflows. Additionally, we offer generalised Bayesian filtering and the hierarchical Gaussian filter as key examples of dynamic networks implemented in our library. The source code, tutorials, and documentation are hosted under the main repository at https://github.com/ComputationalPsychiatry/pyhgf.

## Author summary

Many theories of brain function propose that perception and learning arise from Bayesian inference, in which the brain continuously updates beliefs about the causes of sensory inputs. One influential framework implementing this idea is predictive coding, where hierarchical networks exchange predictions and prediction errors to update internal beliefs. These models have been widely used to study learning, decision-making, and psychiatric conditions. However, applying predictive coding to complex models or large datasets remains difficult because existing tools are often specialized, inflexible, or poorly integrated with modern machine-learning and probabilistic programming frameworks. Here we introduce pyhgf, a software library for building and analysing dynamic predictive coding networks. The library implements the generalised hierarchical Gaussian filter, a model that represents how beliefs and their uncertainty evolve over time. In pyhgf, networks are composed of modular nodes that exchange predictions and prediction errors through local computations, making it possible to flexibly construct complex hierarchical models. The library is designed to integrate with modern computational tools, enabling efficient simulation, parameter estimation, and model comparison. By lowering the technical barriers to building predictive coding models, pyhgf provides a practical framework for studying adaptive behaviour and developing biologically inspired artificial agents.

## 1 Introduction

Bayesian models of cognition describe perception and behaviours as probabilistic inference over the cause of sensory inputs [1]. Scaling these models to infer computational parameters from human behaviours [2–4], or to implement them into artificial agents [5,6], is currently a challenge that brings together computational neuroscience and artificial intelligence. However, when considering living organisms, the complexity of models rapidly increases, and inference becomes especially challenging. While certain inferential processes can sometimes be straightforwardly described and implemented using closed-form solutions, intractability emerges rapidly with models that incorporate multiple information streams, continuous inputs, or hierarchical dependencies common in biological systems. Predictive coding [7,8] proposes that such generative models that support learning and perception can be implemented as hierarchical networks of neurons that exchange predictions and prediction errors through local message passing [9–11]. This mechanism could represent a faster, energy-efficient, and biologically plausible mechanism that could approximate Bayesian inference [9] or gradient-based learning in neural networks [12–14].

One limiting factor to the wider application of predictive coding neural networks to more complex probabilistic models is the absence of easily accessible open-source toolboxes compatible with modern probabilistic programming and neural network libraries. It is therefore critical for the field to develop frameworks that could facilitate their implementation, much as TensorFlow [15] and PyTorch [16] have shaped the development of conventional neural networks. However, this requirement comes with considerable technical challenges on the software development side. Biological systems implement learning and flexible behaviours not only by adjusting inner representations, but also by leveraging self-monitoring, self-organisation, cellular growth, and functional plasticity. Even abstracting from the specific optimisation algorithm or inference method that we aim to apply, only few of these features could be implemented in currently available packages. First, modern frameworks rely on compilation to low-level languages and on automatic differentiation, which often restricts dynamic manipulation of internal variables during execution. For example, conventional neural networks rely heavily on linear algebra functionalities that require static matrix shapes, which has been a limiting factor for graph neural networks (see how [17] and [18] circumvent parts of this problem). Secondly, these frameworks tend to disentangle the optimisation process from the optimised system. While the network is defined through a set of variables only partially transparent, tuning the network relies on the execution of scripts whose steps are hidden from the network, preventing it from reasoning about inference itself. It is therefore crucial, if we wish to adhere to greater biological realism, that future implementations are not affected by such constraints.

A second limitation concerns the possibility to “*observe the observer*” [19] by inferring the parameters of cognitive models from observed decisions and behaviours. Achieving this requires exposing internal variables to statistical inference tools capable of fitting models to large datasets, such as multilevel experimental designs. In practice, this often involves a second inversion of the model using sampling-based inference methods such as Hamiltonian Monte Carlo [20], requiring automatic differentiation of the likelihood function, which is not possible out-of-the-box in several programming languages.

Finally, a last limiting component is the possibility for non-experts to compose custom solutions and networks fitting a restricted domain or task from the collection of available methods. Predictive coding is a family of inference schemes that share the same objective algorithms, which is to approximate Bayesian inference by minimizing variational free energy under mean-field and Laplace assumptions [21,22]. Several computational implementations of this idea have been proposed across different fields [22,23]. Some focus on biophysical and neurobiological realisms [11], while another common class discussed in machine learning performs inference through iterative relaxation dynamics: neuronal activities evolve until the network reaches an equilibrium that minimizes a prediction-error energy function [14]. Toolboxes have been developed to train [24] and benchmark [25] these models. But they also have the downside of having a more limited representation of time resolved uncertainty or volatility (but see also [26]), and rely on gradient-descent dynamics that introduce computational overhead compared to backpropagation [14,21,25].

The *hierarchical Gaussian filter* (HGF) [27,28] provides a complementary formulation tailored to hierarchical dynamical systems. Rather than modelling inference as a relaxation process over neural activities, it derives analytic update equations for the posterior means and precisions of latent states over time [29]. These updates can be interpreted as a predictive-coding scheme in which precision-weighted prediction errors propagate through a hierarchy of Gaussian beliefs. By explicitly representing uncertainty and volatility, the model allows learning rates to adapt dynamically to environmental change. Over the past decade, the HGF has become widely used in computational psychiatry and reinforcement learning to model belief updating in agents operating in changing environments [3]. Many complex cognitive phenomena (e.g., hallucinations and delusions) and psychiatric conditions (e.g., anxiety, autism, schizophrenia) can efficiently be described by alteration of uncertainty or precision processing [30–33]. An important factor in this popularity was the availability of a Matlab toolbox [34], together with its documentation and a forum for community support (https://github.com/ComputationalPsychiatry/hgf-toolbox). This toolbox implements core components (i.e., the two-level and three-level binary and continuous HGF, along with several variations thereof, and an array of response functions). However, generalisation of the model to arbitrarily sized networks [29] is not provided, and the programming language does not allow for interfacing with other Bayesian modelling and neural network tools.

In this paper, we introduce pyhgf, a neural network library for creating, manipulating, and sampling dynamic neural networks for predictive coding. In pyhgf, each local computation is an in-place function operating on the network itself, defined by its attributes, edges, transformations, and propagation dynamics. All network components are modular and transparent during propagation, which means that they can be part of the inference process. It natively supports the implementation of the *generalised hierarchical Gaussian filter* (gHGF) [29], a fully nodalised neural network structure where belief nodes can be flexibly added or removed without any additional derivations. This step considerably extends the complexity of the networks that are supported without requiring additional work from the user and only involves local computations of prediction, prediction error, and posterior updates, as per predictive coding standards. pyhgf is written on top of JAX [35], an XLA and autograd tensor library for Python that supports parallelisation on GPUs and TPUs, as well as in Rust [36], a general-purpose programming language designed for performance and concurrency. The user can decide which of these two backends to use depending on the type of application. This feature allows flexible and computationally efficient network representation, together with smooth integration with other optimisation libraries in the ecosystem [37], both for Bayesian inference (e.g., to iterate HGF models as part of multilevel Bayesian networks) or to interface with other reinforcement learning and neural network libraries [38].

The rest of the paper is organised as follows: we first describe dynamic neural networks from a theoretical and programming point of view, with a focus on the generalized hierarchical Gaussian filter for predictive coding [29], which is a specific instance of such a network. We highlight key differences both with previous versions [27,28] and other software implementations [34]. In the results section, we illustrate the standard workflow supported by the toolbox, from network development to observing the observer. We implement the classical three-level hierarchical Gaussian filter for binary inputs, and demonstrate forward fitting, multiple response models, parameter recovery, and model comparison. Finally, we discuss how the proposed tool could facilitate the creation and simulation of autonomous agents that dynamically approximate high-dimensional distributions to navigate their environment, and highlight new research lines at the interface between computational neuroscience and artificial intelligence.

## 2 Design and implementation

pyhgf is a Python library for the creation, manipulation, and inference over dynamic neural networks for predictive coding with a focus on the generalised hierarchical Gaussian filter [27–29]. Models and theories that imply such networks are becoming ubiquitous in computational neuroscience, and researchers interested in fitting behavioural data to these models require the flexibility of a regular neural network library together with the modularity of a probabilistic framework to perform inference on parameters of interest. In the pyhgf package, we provide the user with an API that provides methods for smoothly interacting with the following two levels of modelling:

1. A set of core methods to define, manipulate, and update dynamic neural networks for predictive coding. These networks need to provide unique flexibility in their design, which is enabled by giving the user control over a limited set of parameters, accessible both to the user in the design process and to the agent in real-time adaptive behaviours.
2. Higher-level classes for embedding any of these networks as custom likelihood functions in multi-level Bayesian models, or as loss functions in other optimization libraries. Those classes include fully defined probabilistic distributions that integrate with PyMC [39] and tools to help diagnose inference, visualization, and model comparison [40].

By using these interfaces, the user is able to customize the computational structure of artificial agents to fit a broad range of applications, both in experimental cognitive neuroscience and artificial intelligence. Here, we start by reviewing the core principles on which dynamic neural networks are built in pyhgf, and how this differs from other network libraries.

### 2.1 Computational framework

The design and software implementation of dynamic neural networks for predictive coding have been shaped by a set of requirements. These networks are made of nodes that can store any number of variables. Some variables might be found in other nodes as well, and some might be unique. Nodes are connected with each other through directed edges, and there can exist multiple types of connections in the same graph, denoting different interactions between the nodes. Computation steps in the graph typically occur locally between adjacent nodes for prediction and prediction errors. Multiple types of computation can be defined. The computation steps can be triggered either reactively, by observing events in the surroundings of a node and reacting to them, or they can be scheduled by pre-allocating a sequence of steps that will propagate the information through the graph. Finally, all these components should be transparent to the network itself when performing a given computation, allowing it to meet the demands of self-monitoring and self-organisation principles.

To observe the set of constraints above, computations should follow a strict functional programming framework, meaning that they should be in-place programmatically pure functions operating on the components of the network. Functional programming is natively supported in Rust and also enforced by JAX [35] to leverage just-in-time (JIT) compilation and automatic differentiation, therefore departing from object-oriented programming (the definition of classes populated with attributes and methods) that is a central feature of Python. This comes with limitations in the way toolboxes’ APIs can be developed (see, for example, how this can be handled in [41]). To fully meet the dynamic aspects mentioned previously, the update functions should ideally receive and return all of the following components defining a network:

1. A list of attributes - the attributes are dictionaries of parameters of a given node
2. A set of lists of edges - the edges are the directed connections between the nodes. All the possible edge types are grouped into a set.
3. A set of update functions. Each function defines a computation and can be parametrised by the index of the triggering node. Possible computations are, for example, prediction, posterior update, or prediction error between two nodes.
4. Unless using a reactive computation scheme, the function should also have access to an ordered sequence of update functions that apply to individual nodes.

**Fig 1:**
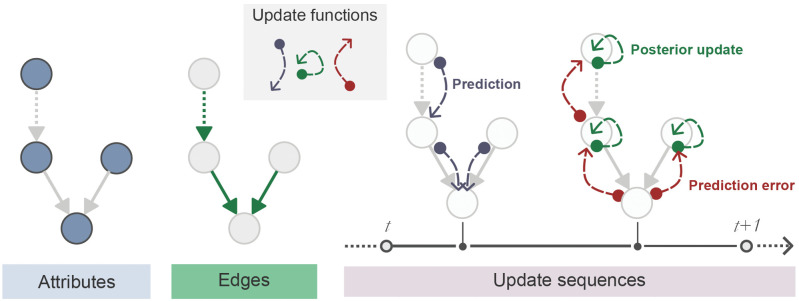
The four components of a dynamic network for predictive coding. pyhgf represents any dynamic network using the combination of four variables: attributes, edges, update functions, and update sequences. This modularity allows dissociating update steps and connectivity structures and makes these variables part of the inference process. The creation of a network is read from left to right: **1. Attributes.** Nodes in the network contain parameters (e.g., sufficient statistics about probability distributions and coupling weights). **2. Edges.** Nodes can have multiple connection types with each other (e.g., value and volatility coupling). The network’s structure is represented in an *m*-dimensional adjacency list that encodes the directed connections with other nodes. Here, dotted and filled lines represent different types of connectivity. **3. Updates.** Update functions are deterministic transformations operating locally that can access and modify the four sets of variables at run time. **4. Update sequences.** The update sequence shapes belief propagation. It defines the order in which nodes should be updated when a new observation is presented to the input node(s). By default, prediction propagates from the leaves to the roots of the network, while the interleaved sequence of prediction errors and posterior updates follow the inverted path. Updates can also be triggered reactively in which case the propagation starts with the activation of a proximal node.

By defining these four components, and by creating functions that can receive and return all of them, the user can generate arbitrarily sized and structured dynamic neural networks for predictive coding (see Fig 1). The first two items define what is usually called a graph, with the addition that it can be directed and multilayered. The last two items shape what is central to predictive coding: the schedule or reactive nature of the propagation of information through the network. Because all of these components are transparent during message-passing computations, learning algorithms can be developed to act on them as a way of inference. Acting on the attributes corresponds to standard inference or other learning algorithms like reinforcement learning. Acting on the size of the networks is comparable to structure learning. Acting on the edges can relate to causal inference, and acting on the update functions or their sequence can implement principles from meta-learning (see 5 for more details on possible applications). The hard constraint on transparency of the network component during message passing makes this framework difficult to implement in other graph/neural network libraries (e.g., see [17,18]). Most of these constraints can be met by using a pure JAX implementation [35] while remaining compatible with transformations like JIT and automatic differentiation. However, some advanced use cases of dynamic reshaping and edge manipulation might result in degraded performance or incompatibility with certain transformations. When using Rust [36] as the backend, all constraints can be met with no cost in terms of performance.

A dynamic network, as implemented in pyhgf is thus defined as the combination of four variables (see Fig 1). Let, for example, ${𝒩}_{k}$ be a neural network with *k* nodes. This network handles in a tuple four parameters of interest:


$$\begin{array}{c} {𝒩}_{k}=\{\Theta ,\Xi ,ℱ,\Sigma \} \end{array}$$ (1)

The variable $\Theta =\{\theta_{1},...,\theta_{k}\}$ represents the nodes’ attributes. Attributes can be used to register local information, like the sufficient statistics of a probability distribution as well as the coupling weights with other nodes. This variable can also be arbitrarily extended to include other fixed parameters or results from other update steps. In a convolutional neural network, Θ would, for example, encode the activation strength. Most standard learning models optimize attributes that belong to this parameter space.

The second key parameter, tightly linked to the first one, is the adjacency list $\Xi =\{\xi_{1},...,\xi_{k}\}$ that controls the shape of the network. Each item in this set registers the directed connection between node *k* and other nodes. Networks that exhibit different connectivity structures propagate information differently. The set of directed connections can be multivariate (a node can have different types of connections with other nodes), such as in multilayer networks [42]. For example, the nodalised hierarchical Gaussian filter [29] assumes two kinds of coupling between nodes: value and volatility coupling. Every edge $\xi_{k}$, therefore, contains *m* = 2 sets of node indices in this case, *m* being the adjacency dimension. By comparison, in a standard recurrent neural network, this variable would define the shape of the layers and their connectivity. Critically, in the proposed framework, this variable is transparent to the update function and can be subject to inference and updates.

The third central component is the set of *n* in-place update functions $ℱ=\{f_{1},...,f_{n}\}$ defining a message passing step operating on the network’s parameter set such as:


$$\begin{array}{c} f_{n}^{k}({𝒩}_{K})={𝒩}_{K}^{\prime } \end{array}$$ (2)

In a convolutional neural network, this set of functions would include the linear product of input and weights, as well as the activation functions. In the generalized HGF [29], this includes three kinds of steps: a prediction (based on previous values and any parent nodes), an update step (based on input from child nodes and the prediction), and the computation of a prediction error. The specific computations in each case depend on the type of coupling between parent and child nodes. Note that the function is parametrized by a target node *k* to which it applies. This allows for defined local computation where only information from a subset of adjacent nodes is used, such as in particular mean field approximations.

Finally, a fourth component is introduced to control the scheduling of these update steps over time as $\Sigma =[f_{1}^{n_{1}},...,f_{i}^{n_{k}},f_{i}\in ℱ,n\in 1,...,k]$. This ordered list describes a sequence of functions parametrized on individual nodes. The update order shapes belief propagation. This component is rarely expressed in the form of a parameter in most conventional applications of neural networks, as well as in the previous implementation of the HGF. This sequence is instead scripted outside the network’s closure and, therefore, not accessible during inference or optimisation. In the case of predictive coding neural networks, however, finer control over belief propagation might be requested by the user, of a kind that also offers flexibility in the modification of belief propagation dynamically. When using the HGF as implemented in pyhgf, this scheduling can be generated at runtime from the network structure Ξ, assuming an ideal belief propagation pattern with a cascade of prediction from the leaves to the roots of the network, and another cascade of prediction error / posterior update pair from the roots to the leaves.

The proposed framework is intended to provide the minimal layout required to create dynamic neural networks for predictive coding. It allows users to create and manipulate the scheduling of updates through a network of nodes while keeping the four components of the network available for inference. Contrary to other neural networks that rely on matrix multiplication for learning, our networks implement local computations that are run sequentially to propagate beliefs along connectivity paths. This also offers a clear dissociation between components that can be developed separately. It is, for example, possible to create alternative message-passing algorithms without having to develop an entire library to simulate the networks, and it is possible to implement existing predictive coding frameworks so users can easily apply them to behavioural data. pyhgf natively support the generalized hierarchical Gaussian filter [29], a recent development of the HGF [27,28] into a nodalised version for predictive coding. In this framework, for example, every node in the network represents a probability distribution of a belief about a latent space in the environment. Beliefs are updated through precision-weighted prediction errors coming from nodes in a lower level of the hierarchy and propagated to higher-level nodes. The exact update functions have been derived in their closed form and can work with arbitrary network architectures [29], which makes this model an excellent application of dynamic neural networks as described here. The relatively widespread use of the HGF in computational psychiatry, and the need for advanced Bayesian modelling tools around it, are also good opportunities to extend the original Matlab toolbox [34] by enhancing the modularity and extensibility of the library.

### 2.2 Optimisation and inference

While predictive coding itself originates from fields related to signal processing and information theory [43], the use of predictive coding as a framework for hierarchical inference [7,8] in biological neural networks makes it especially well-suited to fields related to experimental neuroscience and computational psychiatry. In this context, the neural networks are components of a cognitive model of the subject on which the experimenter performs inference (i.e., observing the observer [19]). For example, in the context of the generalized hierarchical Gaussian filter, the user might be interested in inferring the posterior distribution of tonic volatility at different levels of the hierarchy from observed behaviours.

This kind of reverse inference requires the use of techniques like Markov chain Monte Carlo (MCMC) sampling or gradient descent, which involves the evaluation of several instances of a network, as well as the gradient at evaluation, to find parameters maximizing likelihood. In the Matlab HGF toolbox [34], the inference step is implemented using a variant of the BFGS algorithm, which can be difficult to apply in the context of multilevel models, where there is a particularly pressing need for both high performance and the benefits of automatic differentiation. The pyhgf codebase is entirely written in Python and, as of version 0.2.0, can use JAX [35] as a computational backend which can easily deploy code on CPU, GPU, and TPU. JAX offers a rapidly growing ecosystem for machine learning [37] and artificial intelligence that already includes toolboxes that are conceptually related to predictive coding and hierarchical Gaussian filters, such as state-space modelling (https://github.com/probml/dynamax), reinforcement learning [44], neural networks [38,41] or graph neural networks [18]. We leverage the automatic differentiation and just-in-time compilation offered by JAX [35] to let the networks interface smoothly with other optimisation and inference libraries like PyMC [39] that support a large range of sampling or variational methods, including Hamiltonian Monte Carlo methods such as the No-U-Turn Sampler (NUTS) [45], an approach that has proved to be highly efficient when scaling to high-dimensional problems. While dimensionality was not a major concern for individual model fittings, this can become problematic if we want to model group-level parameters, and therefore estimate a large number of networks together with hyperpriors (multilevel modelling). Assessing group-level estimates is a crucial step for studies in computational psychiatry, where gaining insights into computational parameters at the population level can inform further diagnosis and classification. In pyhgf, it is possible to apply multilevel modelling to any dynamic neural network handled by the toolbox. In the next section, we will focus on the development workflow using the standard three-level hierarchical Gaussian filter as an example.

## 3 Results

**Fig 2:**
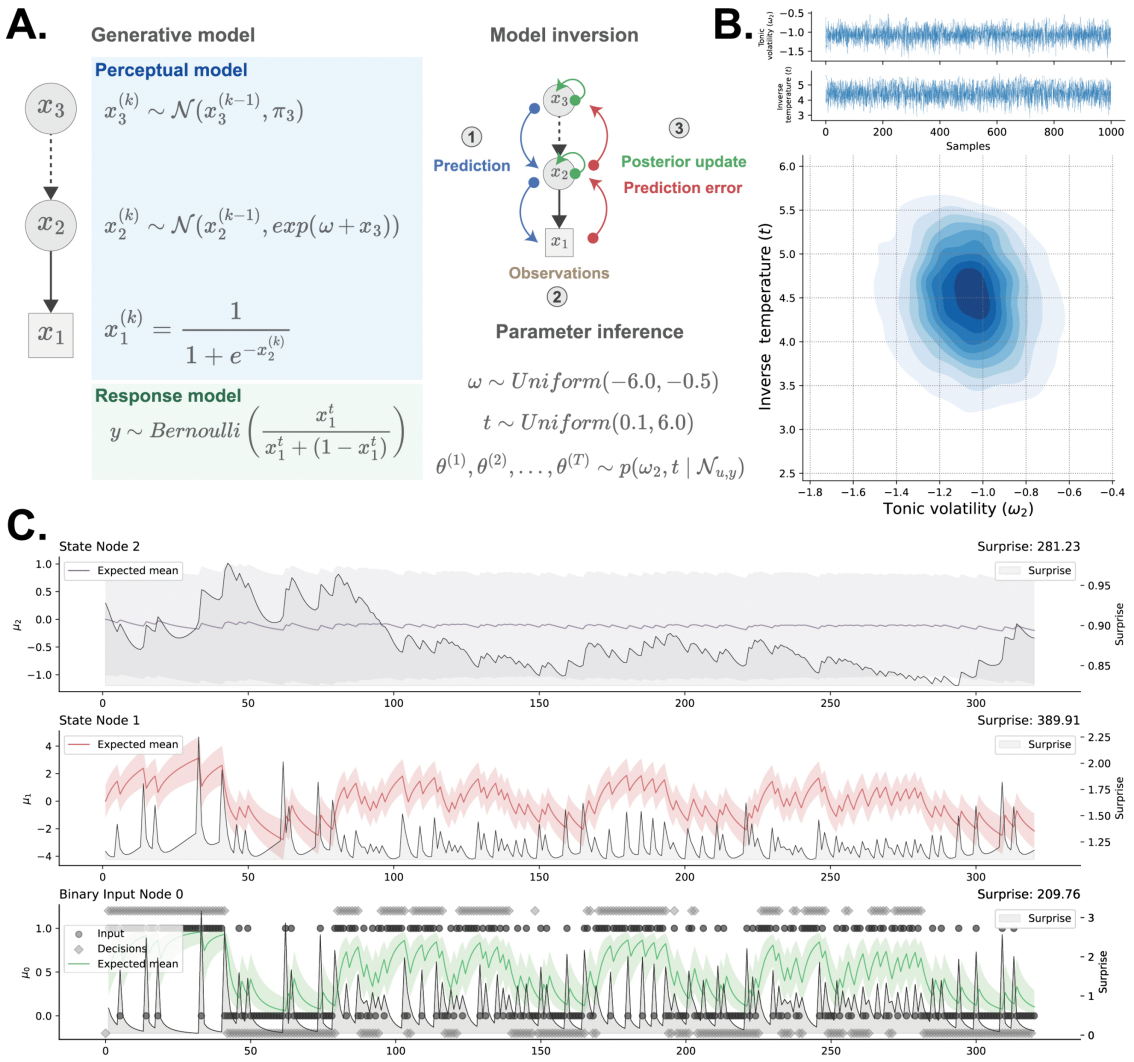
Optimization and inference on the three-level hierarchical Gaussian filters for binary inputs. **A.** 1) Graphical representation of the generative model. Square nodes represent binary state nodes, and circle nodes represent continuous state nodes. Dashed lines indicate volatility coupling while solid lines indicate value coupling. The response model assumes a logistic sigmoid response function with an inverse temperature parameter. 2) Model fitting relies on an inversion of the generative model comprising the top-down propagation of predictions and the bottom-up propagation of prediction errors, driving posterior updates. 3) Parameter inference and optimisation imply a second inversion, namely that of the response model, which relies on automatic differentiation internally. The response function defines the log-probability (or negative surprise) of the observed data under the generative model. **B.** Posterior distribution of inferred parameters. Here, we inferred the value of tonic volatility (ω) at the second level (*x*_2_), and the inverse temperature of the response function **(*t*)**. The upper panel displays the resulting traces (4 chains with 1000 samples), and the bottom panel is a bivariate representation of the corresponding posterior density. All outputs are compatible with PyMC [39], and ArviZ [40] for visualization and diagnostics. **C.** The belief trajectories across time for a model using the best parameter set from the previous steps. The grey-filled curves represent the surprise. The expected mean and precision at each level are depicted using the coloured lines and shaded areas (respectively). The plot was generated using pyhgf’s plotting module.

**Fig 3:**
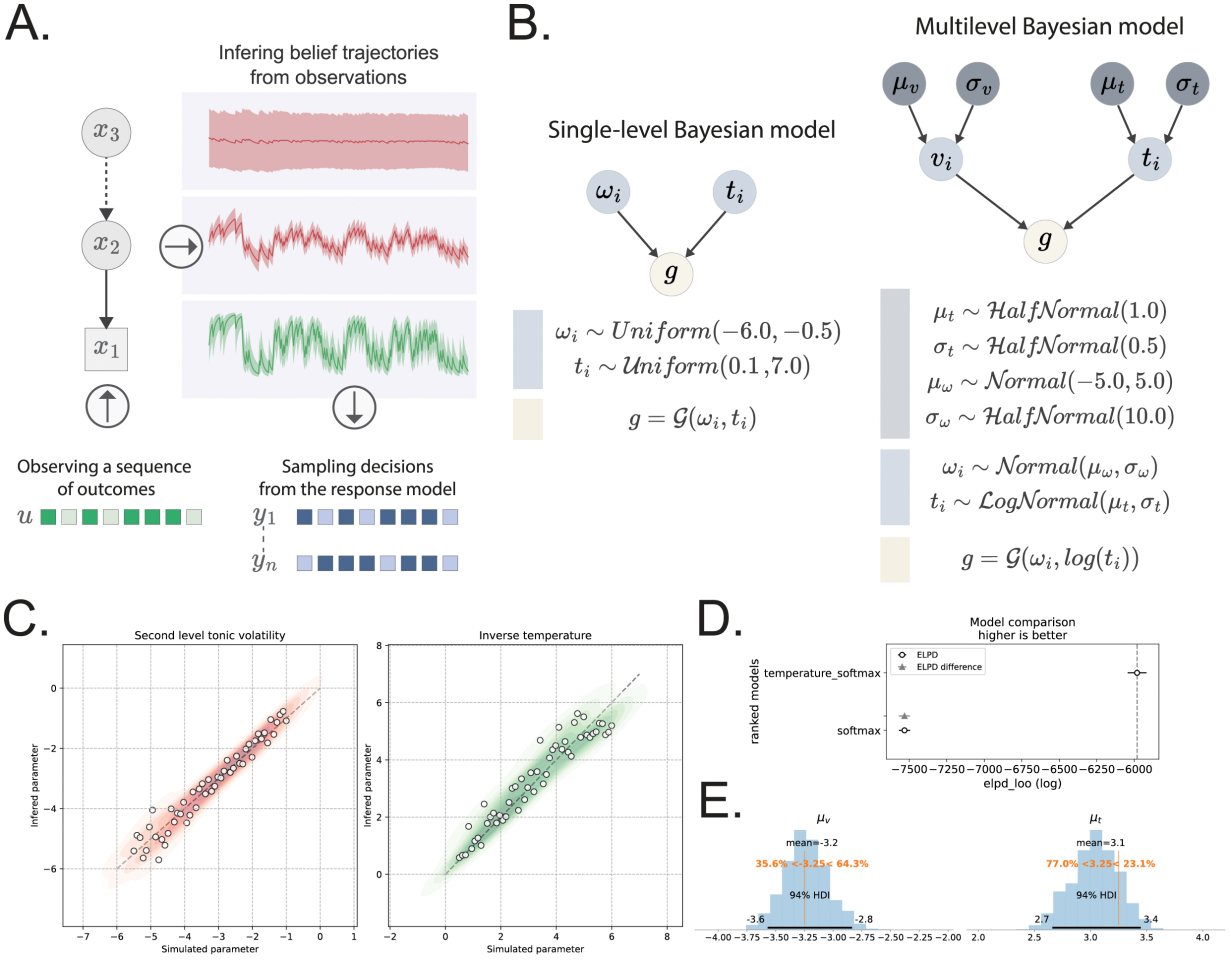
Recovering   computational parameters. **A.** Data simulation. We used a set of observations *u* from [46] as environmental outcomes to simulate belief trajectories under varying values for the second-level volatility (ω) and the inverse temperature **(*t*)**. The expected probability at the first level was then used to sample a vector of decisions using the same response function as described in Fig 2. **B.** Bayesian modelling of the network’s parameters using single-level and multilevel approaches. The single-level approach does not set group constraints on individual parameters and is, in this case, preferred for parameter recovery. We also used this version for the model comparison. The multilevel version puts hyperpriors on top of the individual parameters, enabling inference at the group level. **C.** Parameter recovery. In both panels, the horizontal axis represents the simulated value while the vertical axis represents the recovered/inferred value for tonic volatility at the second level (left) and inverse temperature (right). The dashed line shows the identity line for reference. **D.** Model comparison results. For illustration, we compared a model using a simple sigmoid function as a response function with another one using a sigmoid with an inverse temperature parameter. The plot represents the comparison between the two models based on their expected log pointwise predictive density (ELPD), which is the default recommended method for model comparison when using ArviZ. **E.** Posterior estimates of group-level hyperparameters. Posterior density estimates of group mean for tonic volatility (ω) and temperature **(*t*)**. The orange vertical lines represent empirical group means, the intervals represent the 94% highest density intervals (HDI).

Users interested in using pyhgf are referred to the documentation at https://github.com/ComputationalPsychiatry/pyhgf, which provides a theoretical overview, API descriptions, and tutorials. Here, we illustrate the standard analytic workflow: creating and manipulating dynamic networks, fitting them to a sequence of observations, and performing inference and optimisation over parameters (see Fig 2) relevant for signal processing and real-time decision-making. Parameter estimation in multilevel models, model comparison, and parameter recovery are illustrated in Fig 3 using simulated data.

For the results reported in Fig 2 and in Fig 3, we used binary observations and binary decision responses (hereafter denoted *u* and *y*, respectively) from an associative learning experiment [46]. Such data are well-suited for the binary hierarchical Gaussian filter, so we employed the three-level version of this model as it integrates the core component of the framework. Models were created and visualised using pyhgf v0.2.9. Bayesian inference was performed using PyMC v5.16.2 [39]. Posterior densities and traces were plotted using ArviZ v0.19.0 [40]. The Jupyter notebook used to produce models, figures, and analyses can be retrieved at https://github.com/ComputationalPsychiatry/pyhgf/blob/paper/docs/paper.ipynb.

### 3.1 Generative model, forward fitting and parameter inference

The standard workflow begins by constructing a Bayesian network representing the agent’s generative model of the environment (Fig 2A left panel). In pyhgf, this structure is defined using the Network class, which stores nodes, parameters, edges, and belief update sequences. The resulting graph can be interpreted as a Bayesian network for forward inference (prediction) and used to simulate belief dynamics.

**Listing 1.** Creating a network

from pyhgf.model import Network

*# create a new network* --------------------------------

*# This structure is known as the three-level binary HGF*

binary_hgf = (

Network()

.add_nodes(kind="binary-state")

.add_nodes(kind="continuous-state", value_children=0)

.add_nodes(kind="continuous-state", volatility_children=1)

)

*# visualisation utility to inspect the network’s structure*

binary_hgf.plot_network()

In most applications, however, the model is fitted to observations. Model inversion proceeds through iterative belief propagation at each time step: (1) predictions are propagated from leaves to the root of the network, (2) a new observation is incorporated, and (3) prediction errors propagate upward to update posterior beliefs (Fig 2A right panel). The resulting belief trajectories depend on node attributes such as the tonic volatility at the second level (ω), which modulates the precision of the implied normal distribution and thus the effective learning rate.

Observations are inserted at the first node (*x*_1_), whose inferred mean ($\mu_{1}$) corresponds to the probability of observing category 1. This value is predicted by a continuous node (*x*_2_) representing the logit-transformed probability. A third node (*x*_3_) controls volatility through volatility coupling, allowing fluctuations in *x*_2_. If volatility is assumed constant, the third node can be omitted, resulting in a two-level binary HGF. The internal plotting utilities allow convenient inspection of network structure and debugging, and will produce a figure similar to Fig 2C.

**Listing 2.** Belief trajectories

from pyhgf import load_data

u, y = load_data("binary")

*# provide a vector of binary observations to the network*

binary_hgf.input_data(input_data=u)

*# visualisation utility to inspect beliefs trajectories*

binary_hgf.plot_trajectories()

We provide a sequence of observations using the same class. Fitting the model automatically triggers iterative calls of the belief propagation update. We log the network’s state at each time point in so-called belief trajectories, which describe the agent’s inferred states over time. To evaluate model fit to behaviour, we compute the Bayesian surprise of observed responses using a response function that maps beliefs to actions.

**Listing 3.** Surprise

from pyhgf.math import binary_surprise

import jax.numpy as jnp

def binary_softmax(

hgf,

response_function_inputs,

response_function_parameters,

):

*“”“Surprise under the binary softmax model.”””*

*# the expected values at the first level of the HGF*

beliefs = hgf.node_trajectories[0]["expected_mean"]

*# the binary surprises*

surprise = binary_surprise(x=response_function_inputs, expected_mean=beliefs)

*# ensure that inf is returned if the model cannot fit*

surprise = jnp.where(jnp.isnan(surprise), jnp.inf, surprise)

return surprise

*# compute the sum of the binary surprise*

*# lower values indicate that the model is a better fit to the participant’s behaviour*

binary_hgf.surprise(

response_function = binary_softmax,

response_function_inputs = y

).sum()

The response function specifies the likelihood of observed decisions given the inferred beliefs, and the resulting surprise corresponds to the negative log-probability of the data under the model [19]. We now have all the ingredients to infer parameters governing belief dynamics or decision noise by providing an MCMC sampling algorithm with information about our custom distribution. This interface can be handled automatically using the HGFDistribution class, which will create the relevant structure for sampling inside a PyMC model [39].

**Listing 4.** MCMC sampling

from pyhgf.distribution import HGFDistribution

import pymc as pm

*# create a custom distribution that can be plugged into a PyMC model*

hgf_logp_op = HGFDistribution(

n_levels = 2,

model_type = “binary”,

input_data = u[jnp.newaxis,:],

response_function = binary_softmax,

response_function_inputs=y[jnp.newaxis,:]

)

*# create a PyMC model with one uniform prior over the tonic volatility*

with pm.Model() as two_levels_binary_hgf:

*# Set a prior over the tonic volatility at the second level.*

tonic_volatility_2 = pm.Uniform("tonic_volatility_2", -3.5, 0.0)

*# Call the pre-parametrized HGF distribution here.*

*# All parameters are set to their default value, except omega_2.*

pm.Potential(“hgf_loglike”, hgf_logp_op(tonic_volatility_2 = tonic_volatility_2))

*# visualisation utility to inspect the PyMC computational graph*

*# note that while this is also a network, this is only related to the sampling procedurepm.model_to_graphviz(two_levels_binary_hgf)*

*# sample*

with two_levels_binary_hgf:

two_level_hgf_idata = pm.sample(chains=4, cores=1)

The procedure above describes the perceptual model and explains how beliefs evolve in the network as new observations are made. We then assume that an agent uses available beliefs at time *k* to inform decisions and actions. How to convert beliefs into actions depends on the problem we try to solve. We assume that decisions are generated from the inferred probability $\mu_{1}$ through a logistic sigmoid response function parameterised by an inverse temperature parameter *t*. By estimating parameters such as ω and *t*, we obtain the posterior density $P(\omega_{2},t|{𝒩}_{u,y})$ through MCMC sampling (Fig 2B). The posterior means can then be used *t*o refit the model and recover the belief trajectories most consistent with the observed behaviour (Fig 2C). Because belief updates rely on closed-form variational updates, model inversion is deterministic given fixed inputs and parameters.

### 3.2 Bayesian multilevel modelling, parameter recovery and model comparison

Experiments typically involve multiple participants, requiring joint inference over multiple parameter sets. To illustrate this scenario, we simulated responses for 50 participants using the same observation sequence *u* but different parameter values for ω and *t* (Fig 3A). For each participant, we generated a response vec*t*or $y_{i}$ from the response model and fitted the HGF using the procedure described above.

Since participant fits are independent, the inference can be performed either iteratively or jointly in a single-level Bayesian model (Fig 3B). Parameter recovery was assessed by comparing simulated and inferred values of ω and *t*. As shown in Fig 3C, recovered parameters closely followed the identi*t*y line, indicating reliable recovery from the behavioural data.

Alternative generative models can also be compared on the same dataset, for instance, by modifying network structure or response functions. Here we compared two models differing only in the response function: one with a fixed inverse temperature (*t* = 1) and one in which *t* was free and inferred. Model comparison was performed using leave-one-out cross-validation (LOO) implemented in ArviZ [47]. As expected, the model estimating *t* achieved a higher expected log pointwise predictive density (ELPD) (Fig 3D). Such comparisons should primarily be guided by theoretical considerations and complemented by prior predictive checks and posterior predictive validation.

Finally, population-level inference can be performed using multilevel models in which individual parameters are drawn from group distributions (Fig 3B). This hierarchical approach enables estimation of population parameters and increases statistical power for group comparisons. Panel E. in Fig 3 shows posterior densities of the group means for ω and *t*, together with empirical group means. In bo*t*h cases, the empirical means fall within the 94% highest density interval, indicating reliable population-level estimation.

## 4 Availability and future directions

Bayesian models of cognition have been around for decades, and frameworks like predictive coding are widely used to model information processing in the central nervous system [9]. Their appeal lies in the simplicity and modularity of the computations underlying belief updating, which can support learning and optimization without relying on gradient-based training [13,14], and extend to a variety of domains such as causal inference [48], graph learning [49], or temporal prediction [26].

Here, we introduced pyhgf, a Python library for constructing, manipulating, and sampling dynamic predictive coding networks. Unlike conventional neural network architectures, the networks implemented in pyhgf update their internal representations through local belief propagation rather than through external optimization routines. The framework is intentionally modular and agnostic with respect to the mathematical formalism used for inference and learning. We provide implementations of generalized Bayesian filtering [5] and the generalized hierarchical Gaussian filter [27–29], two important tools for predictive coding.

Networks in pyhgf are defined as rooted trees whose nodes perform simple local update operations. Each update step is implemented as an in-place function operating directly on the network object. This design enables structural plasticity during belief propagation and supports flexible experimentation with network architectures. By separating the computational framework from specific experimental models, the library aims to facilitate methodological development while remaining accessible to users without extensive expertise in predictive coding theory.

**Fig 4:**
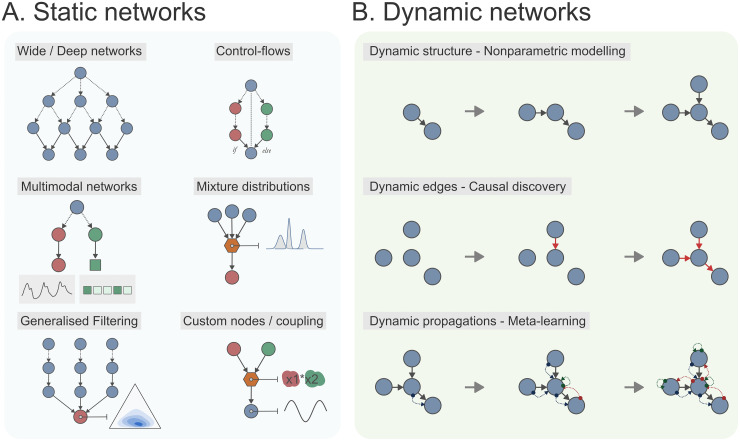
Possible use cases of static and dynamic graphs. **A.** The library supports arbitrary network structure, including deep/wide networks and multivariate dependencies/ascendencies that can handle a large number of inputs with nested hierarchical dependencies (*top-left*). Belief propagation dynamics are adaptable and can implement regular control-flow statements, for example, to condition message passing on the outcome of some assertion (*top-right*). Any node can observe new inputs. Branches of a network observing some inputs will specialise in their dynamics but can share volatility or value at higher levels with other branches (e.g., physiological signals and binary outcomes, *middle-left*). Nodes can capture influence from multiple parents as mixture distributions for online clustering *middle-right*. Any exponential family distribution can be filtered; here, each node traces one sufficient statistic parameter, *bottom-left*. The update steps can be adapted and implement custom operations either at the node level or through coupling functions *bottom-right*. **B.** Dynamic networks can update nodes’ attributes and their structure, edges, and propagation functions. This flexibility can be used to add an inference process that can accommodate catastrophic prediction errors and protect inference in the long term. This can imply increasing the model complexity, a principle known as Bayesian non-parametric modelling, *top*. Adapting the network’s edges can change the causal relationship between variables, a process known as causal discovery *middle*. The propagation dynamic can expand its affordances and become more complex to improve the inference algorithm as new observations are made, a process that borrows principles from meta-learning *bottom*. All the examples here depict networks assuming the context of a generalised Hierarchical Gaussian filter [29], but the principles can easily be adapted to other predictive coding frameworks.

In Sect 3, we illustrated typical workflows using the three-level HGF, a model widely used in computational psychiatry. Here, we discuss broader methodological opportunities enabled by the framework. In particular, the approach allows researchers to explore two complementary regimes: networks with fixed computational graphs and networks whose structure can adapt dynamically during inference (Fig 4). These examples illustrate potential research directions rather than established empirical applications, highlighting how dynamic predictive coding networks may support new hypotheses about learning and inference.

### 4.1 Generalised Bayesian filtering in static networks

Predictive coding models can be viewed as dynamic implementations of Bayesian networks in which belief updates occur through prediction-error-driven message passing [21,22,29]. Even when the network structure is fixed, this formulation provides a flexible way to approximate variational inference in complex generative models [9,50]. One advantage is that arbitrary-sized neural networks can represent arbitrary complex generative models without having to rethink an entire optimisation algorithm. While nodes are implicitly tracking one parameter value through unidimensional normal distributions, more complex probabilistic models can be constructed by combining nodes that track the sufficient statistics of any exponential-family distributions [5], including multivariate cases. This approach in itself offers considerable modularity in real-time probabilistic modelling, as the development of complex variational update algorithms can be replaced by the manipulation of nodes in a large network. Such flexibility may extend the use of the HGF and related predictive coding filters to a broader range of real-time probabilistic modelling problems [51,52].

One straightforward consequence is that networks can be expanded along multiple dimensions: horizontally (by adding more input nodes), vertically (adding more parent nodes), or using multivariate dependencies in which nodes can influence multiple descendants or receive input from multiple parents (see Fig 4A). These extensions allow the modelling of richer generative structures, including mixtures of distributions or custom coupling functions between variables. One straightforward application is the training of deep neural networks to solve prediction and classification tasks (e.g., MNIST or CIFAR), such as what has been implemented in the machine learning literature [53–55]. While work still needs to be done to port the existing predictive coding framework to such an application, this direction is now enabled by our framework. But another potential application is the integration of multimodal data streams frequently encountered in cognitive neuroscience. For example, separate branches could model physiological signals (e.g., respiration, heart rate, EEG, or fMRI) and behavioural outcomes while sharing higher-level volatility estimates. Such models provide a principled way to capture interactions between behavioural and physiological processes within a single generative framework, rather than analysing these modalities separately.

### 4.2 Structure learning, causal inference, and meta-learning in dynamic networks

Beyond flexible connectivity, pyhgf also allows structural variables to be modified during belief propagation. Predictive coding updates are driven by precision-weighted prediction errors, and very large errors may destabilise inference if the model structure is overly restrictive. Dynamic reconfiguration of the network offers an alternative strategy: instead of forcing existing beliefs to accommodate unexpected observations, the model can adapt its structure to better capture the underlying generative process.

This mechanism can naturally express several forms of adaptive learning. For example, nodes can be added or removed during inference, enabling non-parametric model growth in response to unexpected observations (Fig 4B). Similar ideas have recently emerged in research on self-growing neural networks and lifelong learning in reinforcement learning [56,57]. In predictive coding networks, such structural adaptation may support branching, splitting, or merging of model components as environmental complexity changes. Our framework now supports the creation of such networks and behaviours.

Structural adaptation can also target the connectivity between nodes. Because the prediction step of a predictive coding network corresponds to a Bayesian generative graph, modifying edges effectively changes the assumed causal structure between variables. Dynamic adjustment of edges, therefore, provides a natural mechanism for causal discovery from observational data (Fig 4B). Causal inference is increasingly recognised as a core component of biological and artificial learning systems [58–60]. Within the pyhgf framework, causal relations can be inferred in real time, enabling the study of time-varying causal dependencies or volatility coupling on causal edges.

Finally, structural flexibility can extend to the belief propagation dynamics themselves. Since propagation consists of a sequence of update functions, both the sequence and the functions can, in principle, be modified during inference. In pyhgf, update functions are stored as part of the network representation and can therefore evolve as learning progresses. This enables forms of meta-learning in which the system adapts its own inference algorithm to improve predictive accuracy (Fig 4B.). Conceptually, this approach combines ideas from Bayesian non-parametric modelling, where distributions over functions are inferred, with meta-learning approaches in reinforcement learning [61].

In addition to these core computational internal changes, and closer to active inference models, the package also supports the modular extension of the response functions used by the agent (i.e., the beliefs-to-action function). This framework is central for reinforcement learning applications and often requires custom response functions tailored to specific tasks. Examples of how to use custom response functions can be found in the online documentation.

## 5 Conclusion

In this paper, we have introduced pyhgf, a neural network library for predictive coding with a focus on generalized Bayesian filtering and the generalized hierarchical Gaussian filter. We described how the modular definition of neural networks supporting the scheduling of update steps can serve as a generic framework for models relying on the propagation of simple local computations through a hierarchy of layers, such as in predictive coding neural networks. One part of the API is dedicated to the flexible development of dynamic networks, while the second part is oriented towards high-level use and parameter inference, as typically requested for computational neuroscience studies. Together, we hope that this toolbox will help and strengthen the application of predictive neural networks in computational psychiatry, and open new designs in artificial intelligence towards hybrid and complex models of cognition that build on the principled computations derived from predictive coding. pyhgf can be installed from the Python Package Index (https://pypi.org/project/pyhgf/) and the source code is hosted on GitHub under the following public repository: https://github.com/ComputationalPsychiatry/pyhgf. The documentation for the most recent version is accessible at the following link: https://ComputationalPsychiatry.github.io/pyhgf/index.html. The documentation hosts extensive tutorials, examples, and use cases with applications in signal processing, reinforcement learning, and computational psychiatry. We point interested readers to these resources for a deeper practical introduction to the library.

## Supporting information

S1 Text Benchmarking execution time across JAX and Rust with varying networks and input sizes. (PDF)
