# Supplementary material for: pyhgf: A neural network library for predictive coding
Source: PLoS Comput Biol. 2026 Jun 22;22(6):e1014340. doi: 10.1371/journal.pcbi.1014340 (PMC13318038; doi:10.1371/journal.pcbi.1014340)
Supplement: S1 Text — (PDF) [file pcbi.1014340.s001.pdf]

7 Supporting Information

7.1 Execution time of the JAX and Rust backends

We benchmarked the execution time of the JAX and Rust backends across input sequence lengths ranging from 1 to 100,000 timesteps, using both a single 3-node network (a three-level binary HGF) and a wide network consisting of 10 independent copies of the same topology (three-level binary HGF, 30 nodes, 10 parallel input streams). Each configuration was run 30 times after 3 warmup iterations, and we report the mean wall-clock time. For short sequences ($\leq 1,000$ timesteps), the Rust backend was substantially faster, completing a single-network update in 0.004 ms for 1 timestep compared to 1.38 ms for JAX, a roughly 350× speedup attributable to JAX's fixed overhead from XLA dispatch. This advantage persisted across the wide network configuration, where Rust completed in 0.03 ms versus 10.2 ms for JAX at a single timestep. However, as sequence length increased, JAX's XLA-compiled vectorised loop amortised its fixed cost and achieved a lower per-step marginal cost (0.16 μs/step for single, 4.3 μs/step for wide) compared to Rust (0.48 μs/step for single, 9.3 μs/step for wide). The crossover occurred around 10,000 timesteps for the single network configuration, beyond which JAX became faster (3.2 ms vs. 3.7 ms at 10,000 steps; 16.9 ms vs. 48.5 ms at 100,000 steps).

These results suggest that, as of v0.2.9, the Rust backend is best suited for real-time or online filtering applications with short update bursts, while the JAX backend is preferable for batch processing of long time series, where XLA's loop fusion and hardware-optimised linear algebra kernels can be fully exploited. It should also be noted that future optimisation on both sides in future releases (e.g., parallelisation) might change this ratio, where performances are expected to align in the long run as Rust and JAX are compiling to lower-level representations with comparable performances.

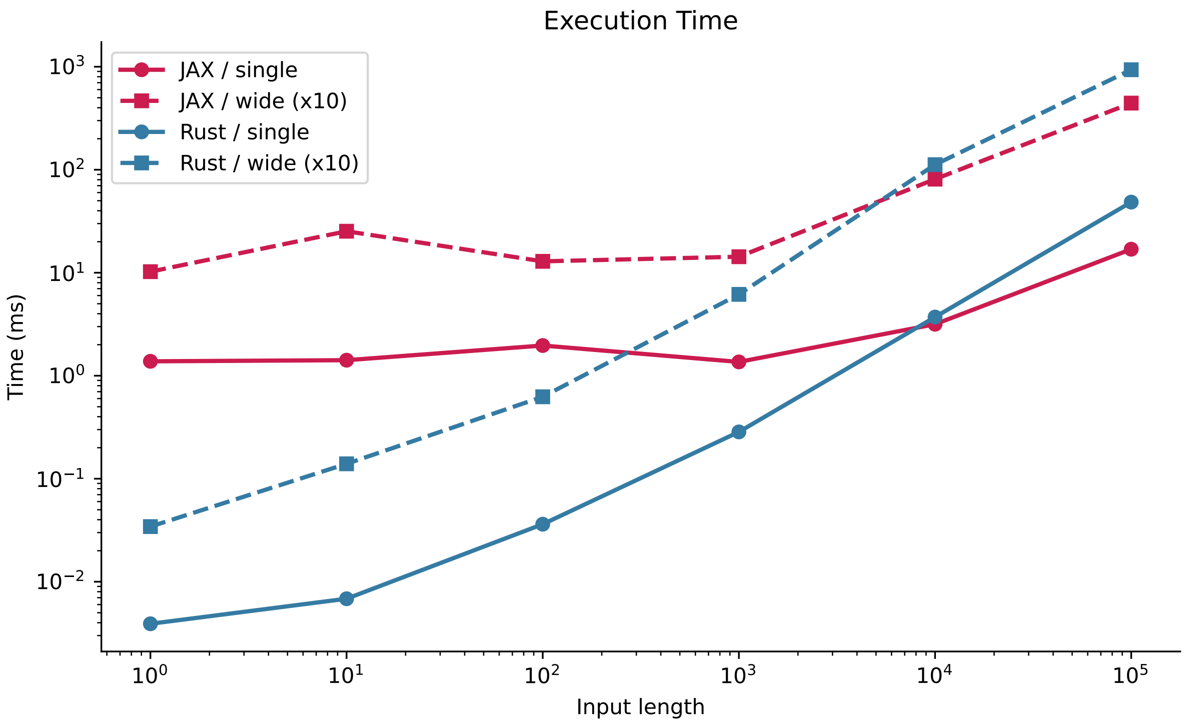

Figure 5. Comparison of the Rust and JAX backend across input and network sizes in `pyhgf` v0.2.9. We measured execution time at inference, varying input size and network topologies. While some of these network topologies would benefit from parallelization, none of the backends used it in this case. As `pyhgf` is meant to address unusual network topologies, dynamic updates, and structure manipulation at inference time, our aim here was to compare the frameworks on these edge cases. This put JAX in a position where it was not able to get most of XLA dispatch, at least for small input sizes. While JAX is losing by a significant margin on smaller input sizes, it should be noted that the framework offers other critical advantages to the user, like automatic differentiation and a simple Numpy-like API that allows a broad range of users to implement and modify critical parts of the codebase, like the response function. For this reason, this backend is still preferable for many users, and Rust should be favored for embedded applications where automatic differentiation is not critical.
