# Supplementary material for: pyhgf: A neural network library for predictive coding
Source: PLoS Comput Biol. 2026 Jun 22;22(6):e1014340. doi: 10.1371/journal.pcbi.1014340 (PMC13318038; doi:10.1371/journal.pcbi.1014340)
Supplement: Attachment — Submitted filename: Response_to_reviewers.pdf [file pcbi.1014340.s003.pdf]

<u>The authors:</u> We thank the reviewers for their constructive and thoughtful feedback. Their comments provided valuable perspectives that helped us improve both the clarity and the positioning of the manuscript.

After carefully considering the reviews, we identified three major areas where the manuscript could be strengthened:

1. **Clearer exposition of the API and code components of the library, including the addition of code examples.**
2. **A more rigorous evaluation of the framework, including benchmarking and discussion of performance and scalability.**
3. **Improved contextualisation of the present work within the broader literature, particularly with respect to existing predictive coding libraries and related frameworks.**

We agree that addressing these points substantially improves the manuscript. Accordingly, we implemented the following revisions:

- **To address Point (1), we expanded the *Results* section and added several code snippets that illustrate the main workflow of the library and highlight its modular design.**
- **To address Point (2), we conducted benchmarking experiments comparing the two backends currently supported by the framework (JAX and Rust). The results are reported in a new Appendix section, along with a discussion of computational trade-offs and scaling considerations.**
- **To address Point (3), we substantially revised the Introduction and the Availability and Future Directions sections. These sections now position the generalized HGF and the pyhgf framework more clearly within the broader predictive coding and computational neuroscience literature. We have also incorporated the additional references suggested by the reviewers.**

As a result of these revisions, several sections of the manuscript—particularly the Introduction, Results, and Availability and Future Directions—have been substantially rewritten to improve clarity, contextualisation, and conciseness.

Below, we address each comment in detail.

Reviewer #1: This manuscript introduces pyhgf, a Python-based library for implementing predictive-coding neural networks in JAX. The authors position the software as a flexible framework for developing dynamic neural networks that embody predictive-coding principles, and they provide extensive discussion of biological motivation, model structuring, and Bayesian inference workflows.

The work addresses a clear gap in the computational neuroscience and computational psychiatry tool ecosystem, as I am not aware of any similar library: differentiable, and highly modular, and also able to merge with contemporary probabilistic programming tools. A small caveat: there are two predictive coding library in JAX already, that allow to implement a different formulation of the algorithm, and have a different goal: that is, to simulate Rao and Ballard's formulation of predictive coding (and more general ones) for machine learning and control tasks. The first one is PCX, more suited for neural network training and deep learning, that allows to implement "Equilibrium propagation-like" predictive coding models [1], the second is NGCLearn, more suited for (copy-paste from the GitHub repo): building, simulating, and analyzing biophysical / neurobiological systems, spiking neuronal networks, predictive coding circuitry, and biomimetic (NeuroAI) agents that learn in a biologically-plausible manner [2].

Minor:

I do not think there is any overlap in scope with the library you are proposing, so the existence of these libraries does not influence the novelty of yours. However, I'd like to see a paragraph explaining differences and similarities. In the same way, I'd like to see a small discussion on how the algorithms your library is able to implement relate/differ from the ones often seen in machine learning conferences (a survey can be found in [3], and many references in the already cited [1]).

<u>The authors:</u> We thank the reviewer for highlighting these relevant libraries. We agree that the manuscript should more clearly position the proposed framework relative to existing predictive coding implementations, particularly those developed in the machine learning community.

To address this point, we have added a paragraph in the Introduction discussing existing predictive coding frameworks implemented in JAX, including PCX and NGC-Learn, and clarified how the present framework differs from these approaches. In particular, we emphasize that these libraries primarily focus on predictive coding formulations inspired by the Rao and Ballard model and related equilibrium-based neural network approaches, often in the context of deep learning or control tasks.

In contrast, the pyhgf framework is designed around generalised Bayesian filtering and hierarchical Gaussian filter models, which rely on analytically derived filtering updates rather than equilibrium relaxation dynamics. We now explicitly discuss these conceptual and methodological differences in the revised manuscript and have included the references suggested by the reviewer.

The manuscript provides a thorough conceptual framing of predictive coding and its computational motivations: The software design principles are clearly articulated, particularly the emphasis on modularity, transparency of internal states, and compatibility with inference engines. A very minor comment is that, while the theoretical exposition is clear, some readers less familiar with predictive-coding frameworks may benefit from a more concise conceptual example early in the manuscript. This also relates to what is probably my main concern in terms of exposition: the lack of code examples. The manuscript describes the software conceptually

but does not show code directly in the text. Given that the contribution is a software package, including a very small runnable example would strengthen accessibility for readers.

Minor:

Would it be possible to come up with 2/4 simple code snippets, that can be added in the main body of the manuscript? It can be a running example that gets increasingly complex the more topics are introduced, or anything else that would allow the reader to grasp the simplicity of the library by simply reading the paper.

[1] Pinchetti, Luca, et al. "Benchmarking Predictive Coding Networks--Made Simple." ICLR 2025.

[2] https://github.com/NACLab/ngc-learn

[3] Salvatori, Tommaso, et al. "A survey on neuro-mimetic deep learning via predictive coding." Neural Networks (2025): 108161.

<u>The authors:</u> **We agree with the reviewer that including code examples would make the framework more accessible to readers, particularly those who are less familiar with predictive coding implementations.**

To address this point, we expanded the Results section to include several code snippets illustrating the main workflow of the library. These examples show how to:

- **define and configure a predictive coding network,**
- **perform forward filtering and belief updates,**
- **integrate the framework with inference tools such as PyMC.**

Each code snippet is accompanied by explanatory comments in the text so that readers can follow the computational logic directly from the manuscript.

Reviewer #2: Overview:

The manuscript introduces pyhgf, a computational neuroscience/computational psychiatry codebase that focuses on building dynamic neural networks, especially those based on predictive coding networks. pyhgf codebase is entirely written in Python and, as of version 0.2.0, it makes use of JAX, an XLA tensor computing library that is robust in leveraging GPUs and TPUs in order to maximize the computational speed of the neural network implementation. The codebase provides specific examples of building generative models, forward fitting and parameter inference as well as Bayesian multi-level modelling, parameter recovery, and model comparison. The framework natively supports generalized hgf (which is typically used in the inference of parameters) and can be integrated into existing libraries such as PyMC.

Strengths:

+ The paper identifies real limitations in existing tools for predictive coding (PC) research. The development of neural networks in typical tensor computing libraries is an obstacle to biologically-inspired learning principles. Therefore, the introduction of pyhgf aims to tackle/address this gap.

+ This library can be integrated into PyMC, a popular framework for Bayesian statistical modeling and variational inference (VI) algorithms

+ The paper is well-written overall, with effective use of figures. Clear presentation!

+ The manuscript contains comprehensive examples. Section 3 provides detailed demonstrations with figures, including forward fitting, parameter inference through MCMC, and so on.

+ The library is implemented in modules such that it can be extended. Overall, there is good implementation design.

Weaknesses:

- The codebase and examples rely heavily on small/toy simulated data. It would be much better/appropriate to also demonstrate the library's efficacy on more complex tasks, such those based on MNIST and natural image/video data (CIFAR, etc.), to demonstrate the applicability and robustness of the library.

<u>The authors:</u> We appreciate this suggestion and agree that demonstrating performance on larger machine learning benchmarks can be valuable in many contexts. However, we believe that this comment reflects a lack of clarity on our side regarding the current scope of the framework.

In the generalized Hierarchical Gaussian Filter, belief propagation relies on analytically derived filtering updates rather than equilibrium relaxation dynamics. As a result, the framework is currently best suited to modelling dynamic systems and agent-environment interactions, which are typical in computational neuroscience and computational psychiatry applications.

For this reason, the current implementation is not specifically designed for solving large-scale classification tasks such as MNIST or CIFAR. Instead, it focuses on sequential probabilistic inference in dynamical systems, where hierarchical latent states evolve over time.

To clarify this point, we revised the Introduction and the Availability and Future Directions sections to explicitly state the intended scope of the library and to explain that extending these models toward large-scale machine learning tasks remains an interesting direction for future work, but this will be approached in separate papers.

- Missing performance benchmark comparison with existing, longer-standing/historical implementations (and related work) such as PyMC (Oriol et al., 2023), NGC-Learn (Ororbia & Kifer, 2022; this is also a JAX-based library) and MATLAB HGF toolbox (Fr¨assle et al., 2021). The performance metrics used can include: computational speed, fitting time, memory usage, scalability when the model size increases, and so on.

<u>The authors:</u> We agree that discussing computational performance is important for users of the framework.

To address this comment, we added a new Appendix section reporting benchmarking results comparing the two backends currently supported by the framework: JAX and Rust. This comparison provides a controlled evaluation, as both backends implement exactly the same algorithms and logging procedures.

Direct benchmarking against other implementations (such as the MATLAB HGF toolbox or other predictive coding frameworks) would be more difficult to interpret because differences in logging procedures, data structures, and internal variables can substantially affect performance measurements. For this reason, we focused on comparing the two backends within the same framework.

The results highlight trade-offs between the two implementations. In general:

- **JAX provides greater flexibility and integrates naturally with probabilistic programming frameworks such as PyMC, making it suitable for model inversion and sampling.**
- **Rust provides a lightweight and efficient implementation for forward simulation and embedding in larger systems.**

We added discussion of these trade-offs in the appendix.

- The manuscript does not address computational limitations. For example, how large can the network grow/be? What is the relative computational runtime when a particular kind of network grows in parameters? There should be significant discussion in the manuscript and perhaps accompanying analysis of what the library cannot do (to guide future users and inform what the library is not as well as what it is).

<u>The authors:</u> We agree that discussing computational limitations is important for guiding users of the library.

We therefore expanded the discussion accompanying the benchmarking appendix to describe current limitations of the framework. In particular, the present implementation does not yet support full vectorisation or parallelisation of updates, meaning that belief propagation in deep networks is currently performed sequentially.

This design choice was intentional in order to preserve the flexibility and dynamic structure of the networks, which may include heterogeneous edge types and sparse connectivity. However, this means that users aiming to implement large, densely connected neural architectures may encounter computational limitations.

We now explicitly describe these trade-offs in the manuscript and discuss how future development could extend the framework to support more scalable implementations.

- Some missing references (related, PC-backing literature for the communities that this article is targeting); missing two key reviews (2nd is published/long-established) for PC background for the modeling tasks (and computational neuroscience side of this article/library) that are related to this work (and third is another missing JAX-based PC library not compared/mentioned as well):

** van Zwol, Björn, Ro Jefferson, and Egon L. Broek. "Predictive coding networks and inference learning: Tutorial and survey." arXiv preprint arXiv:2407.04117 (2024).

** Salvatori, Tommaso, et al. "A survey on neuro-mimetic deep learning via predictive coding." Neural Networks (2025): 108161.

** Innocenti, Francesco, et al. "Jpc: Flexible inference for predictive coding networks in jax." arXiv preprint arXiv:2412.03676 (2024).

The authors: **We thank the reviewer for pointing out these relevant references. We have incorporated the suggested works into the Introduction and related sections of the manuscript, where they are now used to contextualize the proposed framework within the broader predictive coding literature and to highlight connections with recent developments in the field.**

As a result of the points I raised above, I recommend (major) revision for the manuscript as some work needs to be done before it is ready for publication/public dissemination.

Reviewer #3: **Summary**

In this article, the authors present a JAX- and Rust-based library for implementing dynamic networks that perform predictive coding (PC) updates, with a particular focus on hierarchical Gaussian filtering (hGF) models. The manuscript first introduces the theoretical foundations, followed by demonstrations using a generalized hGF model.

Overall, the proposed toolbox represents a valuable addition to the set of computational tools available to researchers working with hGF and predictive coding models. While the core ideas are clearly articulated, the manuscript would benefit from further streamlining (see Point 4 below) that enhances its accessibility and impact. Below, I outline several comments that I hope will help improve the manuscript.

Major Comments :

1. Improved description of the library and API components

Although detailed API documentation is understandably delegated to external docs, a methods-oriented paper would benefit from introducing some of the core library components directly in the manuscript. For example, the authors could include pseudocode or a NumPy-like schematic, as is common in machine learning publications, to illustrate the main computational flow. This would enable readers to more easily connect the theoretical framework to its implementation. Additionally, the authors could consider adding and highlighting high-

level APIs that allow users—particularly those without a programming background—to instantiate and run an hGF model with minimal code, broadening the library's accessibility.

<u>The authors:</u> **We agree with the reviewer that the original manuscript did not sufficiently illustrate the core workflow of the library. To address this issue, we expanded the Results section to include concrete code examples demonstrating how to construct and run predictive coding networks using the framework. These examples illustrate the main computational steps and provide a clearer connection between the theoretical formulation and its implementation.**

2. Stronger integration with existing literature

a. There exist other graph-based implementations of predictive coding that are not discussed. For example, the authors could contrast their network with those of [1].

<u>The authors:</u> **We have added this reference as a contextualisation element when discussing future directions.**

b. Several neural-network-based predictive coding libraries are also absent from the discussion. The authors are encouraged to acknowledge and situate their contribution relative to this work, including but not limited to PredNets, Predify, pypc, NGC-Learn, and PrediRep. Doing so would clarify the novelty and situate the present toolbox within the broader ecosystem of PC libraries.

<u>The authors:</u> **We thank the reviewer for suggesting these additional references. We agree that the manuscript should better situate the framework within the broader ecosystem of predictive coding implementations. Accordingly, we expanded the Introduction to include a discussion of several related libraries and clarified how the present framework differs in terms of goals and methodological focus.**

3. Lack of a central conceptual contribution

A key concern with the current manuscript is the lack of a clearly articulated overarching message beyond the presentation of the toolbox itself. While the software is undoubtedly a useful resource, many of the principal conceptual insights appear to have been addressed in the authors' prior publications. Including additional empirical or methodological insights would help strengthen the significance of the work. Given the use of JAX and Rust, maybe the authors could emphasize and quantify the computational advantages that arise from this design choice?

<u>The authors:</u> **We appreciate this comment and agree that the manuscript should better highlight the computational aspects of the framework. To address this concern, we added a benchmarking section in the appendix that evaluates the computational characteristics of the framework under different backends. This analysis provides quantitative insight into the computational trade-offs associated with the design choices underlying the library. See also our response to Reviewer 2 on that note.**

4. Speculative discussion and focus

The discussion surrounding topics such as self-organization, causality, and others is largely speculative and not directly supported by empirical evidence in the manuscript. I would recommend keeping the main text focused on concrete methodological contributions and moving these more speculative considerations to a concise section of the Discussion. Alternatively, providing additional results that directly support these claims would also help address the concern raised in Point 3.

The authors: We understand the reviewer's concern that certain sections discussing topics such as self-organisation and causal discovery may appear speculative in the absence of empirical demonstrations. Our intention in these sections was to provide transparency regarding the design motivations behind the framework and to highlight potential future applications enabled by its architecture. However, we agree that these discussions should not distract from the main methodological contribution of the manuscript. We therefore condensed these sections and moved the more speculative elements into a shorter discussion of future research directions, while expanding the contextualisation of the framework relative to existing predictive coding libraries.

References

1 - Salvatori, Tommaso, Luca Pinchetti, Beren Millidge, et al. 2022. "Learning on Arbitrary Graph Topologies via Predictive Coding." arXiv:2201.13180. Preprint, arXiv, October 12. https://doi.org/10.48550/arXiv.2201.13180.
