# Supplementary material for: pyhgf: A neural network library for predictive coding
Source: PLoS Comput Biol. 2026 Jun 22;22(6):e1014340. doi: 10.1371/journal.pcbi.1014340 (PMC13318038; doi:10.1371/journal.pcbi.1014340)
Supplement: Peer review history

# Peer review: Decision Letter 0

**DOI:** 10.1371/journal.pcbi.1014340.r001

**Authors:** Paul Bays

14 Jan 2026

PCOMPBIOL-D-25-01766

pyhgf: A neural network library for predictive coding

PLOS Computational Biology

Dear Dr. Legrand,

Thank you for submitting your manuscript to PLOS Computational Biology. After careful consideration, we feel that it has merit but does not fully meet PLOS Computational Biology's publication criteria as it currently stands. Therefore, we invite you to submit a revised version of the manuscript that addresses the points raised during the review process.

Please submit your revised manuscript by Mar 16 2026 11:59PM. If you will need more time than this to complete your revisions, please reply to this message or contact the journal office at ploscompbiol@plos.org. When you're ready to submit your revision, log on to https://www.editorialmanager.com/pcompbiol/ and select the 'Submissions Needing Revision' folder to locate your manuscript file.

Please include the following items when submitting your revised manuscript:

* A letter that responds to each point raised by the editor and reviewer(s). You should upload this letter as a separate file labeled 'Response to Reviewers'. This file does not need to include responses to formatting updates and technical items listed in the 'Journal Requirements' section below.

* A marked-up copy of your manuscript that highlights changes made to the original version. You should upload this as a separate file labeled 'Revised Manuscript with Track Changes'.

* An unmarked version of your revised paper without tracked changes. You should upload this as a separate file labeled 'Manuscript'.

If you would like to make changes to your financial disclosure, competing interests statement, or data availability statement, please make these updates within the submission form at the time of resubmission. Guidelines for resubmitting your figure files are available below the reviewer comments at the end of this letter

We look forward to receiving your revised manuscript.

Kind regards,

Paul Bays

Academic Editor

PLOS Computational Biology

Hugues Berry

Section Editor

PLOS Computational Biology

**Additional Editor Comments:**

As you will see, the Reviewers have positive things to say about your manuscript but also raised some concerns that will need to be addressed before a final decision can be made.

**Journal Requirements:**

1) We ask that a manuscript source file is provided at Revision. Please upload your manuscript file as a .doc, .docx, .rtf or .tex. If you are providing a .tex file, please upload it under the item type u2018LaTeX Source Fileu2019 and leave your .pdf version as the item type u2018Manuscriptu2019.

2) Please provide an Author Summary. This should appear in your manuscript between the Abstract (if applicable) and the Introduction, and should be 150-200 words long. The aim should be to make your findings accessible to a wide audience that includes both scientists and non-scientists. Sample summaries can be found on our website under Submission Guidelines:

https://journals.plos.org/ploscompbiol/s/submission-guidelines#loc-parts-of-a-submission

3) Your manuscript is missing the following sections: Results, and Availability and Future Directions. Please ensure that your article adheres to the standard Software article layout and order of Abstract, Introduction, Design and Implementation, Results, and Availability and Future Directions. For details on what each section should contain, see our Software article guidelines:

https://journals.plos.org/ploscompbiol/s/submission-guidelines#loc-software-submissions

4) Please upload all main figures as separate Figure files in .tif or .eps format. For more information about how to convert and format your figure files please see our guidelines:

https://journals.plos.org/ploscompbiol/s/figures

**Reviewers' comments:**

Reviewer's Responses to Questions

**Comments to the Authors:**

**Please note here if the review is uploaded as an attachment.**

Reviewer #1: This manuscript introduces pyhgf, a Python-based library for implementing predictive-coding neural networks in JAX. The authors position the software as a flexible framework for developing dynamic neural networks that embody predictive-coding principles, and they provide extensive discussion of biological motivation, model structuring, and Bayesian inference workflows.

The work addresses a clear gap in the computational neuroscience and computational psychiatry tool ecosystem, as I am not aware of any similar library: differentiable, and highly modular, and also able to merge with contemporary probabilistic programming tools. A small caveat: there are two predictive coding library in JAX already, that allow to implement a different formulation of the algorithm, and have a different goal: that is, to simulate Rao and Ballard’s formulation of predictive coding (and more general ones) for machine learning and control tasks. The first one is PCX, more suited for neural network training and deep learning, that allows to implement “Equilibrium propagation-like” predictive coding models [1], the second is NGCLearn, more suited for (copy-paste from the GitHub repo): building, simulating, and analyzing biophysical / neurobiological systems, spiking neuronal networks, predictive coding circuitry, and biomimetic (NeuroAI) agents that learn in a biologically-plausible manner [2].

Minor:

I do not think there is any overlap in scope with the library you are proposing, so the existence of these libraries does not influence the novelty of yours. However, I’d like to see a paragraph explaining differences and similarities. In the same way, I’d like to see a small discussion on how the algorithms your library is able to implement relate/differ from the ones often seen in machine learning conferences (a survey can be found in [3], and many references in the already cited [1]).

The manuscript provides a thorough conceptual framing of predictive coding and its computational motivations: The software design principles are clearly articulated, particularly the emphasis on modularity, transparency of internal states, and compatibility with inference engines. A very minor comment is that, while the theoretical exposition is clear, some readers less familiar with predictive-coding frameworks may benefit from a more concise conceptual example early in the manuscript. This also relates to what is probably my main concern in terms of exposition: the lack of code examples. The manuscript describes the software conceptually but does not show code directly in the text. Given that the contribution is a software package, including a very small runnable example would strengthen accessibility for readers.

Minor:

Would it be possible to come up with 2/4 simple code snippets, that can be added in the main body of the manuscript? It can be a running example that gets increasingly complex the more topics are introduced, or anything else that would allow the reader to grasp the simplicity of the library by simply reading the paper.

[1] Pinchetti, Luca, et al. "Benchmarking Predictive Coding Networks--Made Simple." ICLR 2025.

[2] https://github.com/NACLab/ngc-learn

[3] Salvatori, Tommaso, et al. "A survey on neuro-mimetic deep learning via predictive coding." Neural Networks (2025): 108161.

Reviewer #2: Overview:

The manuscript introduces pyhgf, a computational neuroscience/computational psychiatry codebase that focuses on building dynamic neural networks, especially those based on predictive coding networks. pyhgf codebase is entirely written in Python and, as of version 0.2.0, it makes use of JAX, an XLA tensor computing library that is robust in leveraging GPUs and TPUs in order to maximize the computational speed of the neural network implementation. The codebase provides specific examples of building generative models, forward fitting and parameter inference as well as Bayesian multi-level modelling, parameter recovery, and model comparison. The framework natively supports generalised hgf (which is typically used in the inference of parameters) and can be integrated into existing libraries such as PyMC.

Strengths:

+ The paper identifies real limitations in existing tools for predictive coding (PC) research. The development of neural networks in typical tensor computing libraries is an obstacle to biologically-inspired learning principles. Therefore, the introduction of pyhgf aims to tackle/address this gap.

+ This library can be integrated into PyMC, a popular framework for Bayesian statistical modeling and variational inference (VI) algorithms

+ The paper is well-written overall, with effective use of figures. Clear presentation!

+ The manuscript contains comprehensive examples. Section 3 provides detailed demonstrations with figures, including forward fitting, parameter inference through MCMC, and so on.

+ The library is implemented in modules such that it can be extended. Overall, there is good implementation design.

Weaknesses:

- The codebase and examples rely heavily on small/toy simulated data. It would be much better/appropriate to also demonstrate the library’s efficacy on more complex tasks, such those based on MNIST and natural image/video data (CIFAR, etc.), to demonstrate the applicability and robustness of the library.

- Missing performance benchmark comparison with existing, longer-standing/historical implementations (and related work) such as PyMC (Oriol et al., 2023), NGC-Learn (Ororbia & Kifer, 2022; this is also a JAX-based library) and MATLAB HGF toolbox (Frässle et al., 2021). The performance metrics used can include: computational speed, fitting time, memory usage, scalability when the model size increases, and so on.

- The manuscript does not address computational limitations. For example, how large can the network grow/be? What is the relative computational runtime when a particular kind of network grows in parameters? There should be significant discussion in the manuscript and perhaps accompanying analysis of what the library cannot do (to guide future users and inform what the library is not as well as what it is).

- Some missing references (related, PC-backing literature for the communities that this article is targeting); missing two key reviews (2nd is published/long-established) for PC background for the modeling tasks (and computational neuroscience side of this article/library) that are related to this work (and third is another missing JAX-based PC library not compared/mentioned as well):

** van Zwol, Björn, Ro Jefferson, and Egon L. Broek. "Predictive coding networks and inference learning: Tutorial and survey." arXiv preprint arXiv:2407.04117 (2024).

** Salvatori, Tommaso, et al. "A survey on neuro-mimetic deep learning via predictive coding." Neural Networks (2025): 108161.

** Innocenti, Francesco, et al. "Jpc: Flexible inference for predictive coding networks in jax." arXiv preprint arXiv:2412.03676 (2024).

As a result of the points I raised above, I recommend (major) revision for the manuscript as some work needs to be done before it is ready for publication/public dissemination.

Reviewer #3: **Summary**

In this article, the authors present a JAX- and Rust-based library for implementing dynamic networks that perform predictive coding (PC) updates, with a particular focus on hierarchical Gaussian filtering (hGF) models. The manuscript first introduces the theoretical foundations, followed by demonstrations using a generalized hGF model.

Overall, the proposed toolbox represents a valuable addition to the set of computational tools available to researchers working with hGF and predictive coding models. While the core ideas are clearly articulated, the manuscript would benefit from further streamlining (see Point 4 below) that enhances its accessibility and impact. Below, I outline several comments that I hope will help improve the manuscript.

**Major Comments :**

1. Improved description of the library and API components

Although detailed API documentation is understandably delegated to external docs, a methods-oriented paper would benefit from introducing some of the core library components directly in the manuscript. For example, the authors could include pseudocode or a NumPy-like schematic, as is common in machine learning publications, to illustrate the main computational flow. This would enable readers to more easily connect the theoretical framework to its implementation. Additionally, the authors could consider adding and highlighting high-level APIs that allow users—particularly those without a programming background—to instantiate and run an hGF model with minimal code, broadening the library’s accessibility.

2. Stronger integration with existing literature

a. There exist other graph-based implementations of predictive coding that are not discussed. For example, the authors could contrast their network with those of [1].

b. Several neural-network-based predictive coding libraries are also absent from the discussion. The authors are encouraged to acknowledge and situate their contribution relative to this work, including but not limited to PredNets, Predify, pypc, NGC-Learn, and PrediRep. Doing so would clarify the novelty and situate the present toolbox within the broader ecosystem of PC libraries.

3. Lack of a central conceptual contribution

A key concern with the current manuscript is the lack of a clearly articulated overarching message beyond the presentation of the toolbox itself. While the software is undoubtedly a useful resource, many of the principal conceptual insights appear to have been addressed in the authors’ prior publications. Including additional empirical or methodological insights would help strengthen the significance of the work. Given the use of JAX and Rust, maybe the authors could emphasize and quantify the computational advantages that arise from this design choice?

4. Speculative discussion and focus

The discussion surrounding topics such as self-organization, causality, and others is largely speculative and not directly supported by empirical evidence in the manuscript. I would recommend keeping the main text focused on concrete methodological contributions and moving these more speculative considerations to a concise section of the Discussion. Alternatively, providing additional results that directly support these claims would also help address the concern raised in Point 3.

References

1 - Salvatori, Tommaso, Luca Pinchetti, Beren Millidge, et al. 2022. “Learning on Arbitrary Graph Topologies via Predictive Coding.” arXiv:2201.13180. Preprint, arXiv, October 12. https://doi.org/10.48550/arXiv.2201.13180.

**********

**Have the authors made all data and (if applicable) computational code underlying the findings in their manuscript fully available?**

The PLOS Data policy requires authors to make all data and code underlying the findings described in their manuscript fully available without restriction, with rare exception (please refer to the Data Availability Statement in the manuscript PDF file). The data and code should be provided as part of the manuscript or its supporting information, or deposited to a public repository. For example, in addition to summary statistics, the data points behind means, medians and variance measures should be available. If there are restrictions on publicly sharing data or code —e.g. participant privacy or use of data from a third party—those must be specified.

Reviewer #1: Yes

Reviewer #2: Yes

Reviewer #3: None

**********

PLOS authors have the option to publish the peer review history of their article (what does this mean?). If published, this will include your full peer review and any attached files.

If you choose “no”, your identity will remain anonymous but your review may still be made public.

**Do you want your identity to be public for this peer review?**  For information about this choice, including consent withdrawal, please see our Privacy Policy.

Reviewer #1: No

Reviewer #2: No

Reviewer #3: No

[NOTE: If reviewer comments were submitted as an attachment file, they will be attached to this email and accessible via the submission site. Please log into your account, locate the manuscript record, and check for the action link "View Attachments". If this link does not appear, there are no attachment files.]

**Figure resubmission:**

While revising your submission, we strongly recommend that you use PLOS’s NAAS tool (https://ngplosjournals.pagemajik.ai/artanalysis) to test your figure files. NAAS can convert your figure files to the TIFF file type and meet basic requirements (such as print size, resolution), or provide you with a report on issues that do not meet our requirements and that NAAS cannot fix.

After uploading your figures to PLOS’s NAAS tool - https://ngplosjournals.pagemajik.ai/artanalysis, NAAS will process the files provided and display the results in the "Uploaded Files" section of the page as the processing is complete. If the uploaded figures meet our requirements (or NAAS is able to fix the files to meet our requirements), the figure will be marked as "fixed" above. If NAAS is unable to fix the files, a red "failed" label will appear above. When NAAS has confirmed that the figure files meet our requirements, please download the file via the download option, and include these NAAS processed figure files when submitting your revised manuscript.

**Reproducibility:**

To enhance the reproducibility of your results, we recommend that authors of applicable studies deposit laboratory protocols in protocols.io, where a protocol can be assigned its own identifier (DOI) such that it can be cited independently in the future. Additionally, PLOS ONE offers an option to publish peer-reviewed clinical study protocols. Read more information on sharing protocols at https://plos.org/protocols?utm_medium=editorial-email&utm_source=authorletters&utm_campaign=protocols

# Author comment: Author response to Decision Letter 1

**DOI:** 10.1371/journal.pcbi.1014340.r002

9 Apr 2026

Attachment Submitted filename: Response_to_reviewers.pdf

# Peer review: Decision Letter 1

**DOI:** 10.1371/journal.pcbi.1014340.r003

**Authors:** Paul Bays

18 May 2026

Dear Mr. Legrand,

We are pleased to inform you that your manuscript 'pyhgf: A neural network library for predictive coding' has been provisionally accepted for publication in PLOS Computational Biology.

Before your manuscript can be formally accepted you will need to complete some formatting changes, which you will receive in a follow up email. A member of our team will be in touch with a set of requests.

Please note that your manuscript will not be scheduled for publication until you have made the required changes, so a swift response is appreciated.

IMPORTANT: The editorial review process is now complete. PLOS will only permit corrections to spelling, formatting or significant scientific errors from this point onwards. Requests for major changes, or any which affect the scientific understanding of your work, will cause delays to the publication date of your manuscript.

Should you, your institution's press office or the journal office choose to press release your paper, you will automatically be opted out of early publication. We ask that you notify us now if you or your institution is planning to press release the article. All press must be co-ordinated with PLOS.

Thank you again for supporting Open Access publishing; we are looking forward to publishing your work in PLOS Computational Biology.

Best regards,

Paul Bays

Academic Editor

PLOS Computational Biology

Hugues Berry

Section Editor

PLOS Computational Biology

***********************************************************

Reviewer's Responses to Questions

**Comments to the Authors:**

**Please note here if the review is uploaded as an attachment.**

Reviewer #1: Dear authors,

I have reviewed the submitted changes, and evaluated them in line with my review and the one provided by the other reviewers. I'm happy with the re-submitted version, and hence recommend acceptance.

Reviewer #3: I would like to thank the authors for their detailed responses. I think the manuscript reads well now and seems fit for publication.

**********

**Have the authors made all data and (if applicable) computational code underlying the findings in their manuscript fully available?**

The PLOS Data policy requires authors to make all data and code underlying the findings described in their manuscript fully available without restriction, with rare exception (please refer to the Data Availability Statement in the manuscript PDF file). The data and code should be provided as part of the manuscript or its supporting information, or deposited to a public repository. For example, in addition to summary statistics, the data points behind means, medians and variance measures should be available. If there are restrictions on publicly sharing data or code —e.g. participant privacy or use of data from a third party—those must be specified.

Reviewer #1: Yes

Reviewer #3: Yes

**********

PLOS authors have the option to publish the peer review history of their article (what does this mean?). If published, this will include your full peer review and any attached files.

If you choose “no”, your identity will remain anonymous but your review may still be made public.

**Do you want your identity to be public for this peer review?**  For information about this choice, including consent withdrawal, please see our Privacy Policy.

Reviewer #1: No

Reviewer #3: No

# Editor report: Acceptance letter

**DOI:** 10.1371/journal.pcbi.1014340.r004

**Authors:** Paul Bays

PCOMPBIOL-D-25-01766R1

pyhgf: A neural network library for predictive coding

Dear Dr Legrand,

I am pleased to inform you that your manuscript has been formally accepted for publication in PLOS Computational Biology. Your manuscript is now with our production department and you will be notified of the publication date in due course.

The corresponding author will soon be receiving a typeset proof for review, to ensure errors have not been introduced during production. Please review the PDF proof of your manuscript carefully, as this is the last chance to correct any errors. Please note that major changes, or those which affect the scientific understanding of the work, will likely cause delays to the publication date of your manuscript.

Soon after your final files are uploaded, unless you have opted out, the early version of your manuscript will be published online. The date of the early version will be your article's publication date. The final article will be published to the same URL, and all versions of the paper will be accessible to readers.

For Research, Software, and Methods articles, you will receive an invoice from PLOS for your publication fee after your manuscript has reached the completed accept phase. If you receive an email requesting payment before acceptance or for any other service, this may be a phishing scheme. Learn how to identify phishing emails and protect your accounts at https://explore.plos.org/phishing.

Thank you again for supporting PLOS Computational Biology and open-access publishing. We are looking forward to publishing your work!

With kind regards,

Zsofia Freund

PLOS Computational Biology | Carlyle House, Carlyle Road, Cambridge CB4 3DN | United Kingdom ploscompbiol@plos.org | Phone +44 (0) 1223-442824 | ploscompbiol.org | @PLOSCompBiol
